# Drug utilization patterns among elderly hospitalized patients on poly-pharmacy in Punjab, Pakistan

**DOI:** 10.1186/s40545-017-0112-z

**Published:** 2017-08-04

**Authors:** Muhammad Rehan Sarwar, Muhammad Atif, Shane Scahill, Anum Saqib, Muhammad Qamar-uz-Zaman, Zaheer Babar

**Affiliations:** 1 Department of Pharmacy, The Islamia University of Bahawalpur, Bahawalpur, Pakistan; 2 School of Management, Massey University, Auckland, New Zealand; 3 Department of Pharmacy, University of Huddersfield, Huddersfield, UK

**Keywords:** Drug use, Drug utilization pattern, Elderly, Hospitalized, Poly-pharmacy

## Abstract

**Background:**

Reports from drug utilization reviews are important tools employed in the assessment of healthcare practices. The objective of this study was to evaluate drug utilization patterns among elderly hospitalized patients on poly-pharmacy regimens in Pakistan.

**Methods:**

A descriptive, non-experimental, cross-sectional study was carried out from December 2015 to March 2016 in six tertiary-care hospitals in the Punjab province of Pakistan. The population under study were patients aged ≥60 years, taking ≥5 medicines per day (i.e., patients on poly-pharmacy) and who were hospitalized in the selected tertiary-care hospitals. In this study, data was collected from 600 hospitalized elderly patients (100 patients per hospital). All medicines prescribed on each in-patient chart were noted on a pre-designed pro-forma sheet and were classified under the Anatomical Therapeutic Chemical (ATC) classification system. Multiple linear regression analysis was used to determine the independent factors associated with poly-pharmacy in this cohort. Statistical Package for Social Sciences (SPSS) was used to analyze the data. *P*-value < .05 indicated statistical significance.

**Results:**

In 600 hospitalized in-patient (male 52.7% and female 47.3%) medication charts, 3179 medicines were prescribed. The most commonly prescribed drug classes were: A: alimentary tract and metabolism 80% (A02: drugs for acid related disorders 64.5%, A03: drugs for functional gastrointestinal disorders 21.5%), N: nervous system 66.3% (N02: analgesics 67.2%, N03: antiepileptic’s 11.2%), J: anti-infectives for systemic use 62.2% (J01: antibacterial for systemic use 82.5%, J04: antimycobacterials 15.3%) and C: cardiovascular system 48.3% (C07: beta blocking agents 19.8%, C10: lipid modifying agents 16.5%), respectively. The most commonly prescribed active substances were: A02BC01 (omeprazole 51.3%), N02BE01 (paracetamol 50.8%) and J01DD04 (ceftriaxone 40.2%), respectively. In multiple linear regression analysis, male gender (95% CI −.205, −.006, *p* = .039, B = −.091), being divorced (95% CI −.604, −.136, *p* = .002, B = −.130) and presence of comorbidity (95% CI .068, .267, *p* = .001, B = .144) were the independent factors associated with increased drug use among elderly hospitalized patients on poly-pharmacy.

**Conclusions:**

The rational use of medicines is of utmost importance, most particularly in the elderly population. More consideration should be given to rationalizing pharmacotherapy in elderly hospitalized patients who are on poly-pharmacy regimens in Pakistan.

**Electronic supplementary material:**

The online version of this article (doi:10.1186/s40545-017-0112-z) contains supplementary material, which is available to authorized users.

## Background

Drug therapy serves as the commonest medical intervention reducing health related risks across numerous diseases [1]. A number of studies have been conducted globally to explore the socio-demographic [2, 3], medical related [3, 4] and health system factors [5] as substantial influencers of drug utilization. Despite this, a limited literature is available on drug utilization within a multivariate framework which considers all of the aforementioned variables [1, 6, 7]; particularly in Pakistan.

The elderly are more prone to chronic illnesses due to aging and physiological changes; with the majority of older people (up to about 80%) suffering from chronic illnesses [8]. Consequently, this group are more likely to have increased drug utilization over the general population [9]. Medical, social and financial changes, both at the individual and societal level, are the consequence of geographical differences and/or changes in medicine use over time, and there is a need to identify, explain and remedy these pharmaco-epidemiological differences.

There is scarce data available on drug utilization among elderly in Pakistan and the investigation of poly-pharmacy (taking ≥5 medicines per day) remains under studied. Elderly people in Pakistan comprise a large proportion drug use and this has led to an increase in total health expenditure. Pakistan’s demographic trends demonstrate that between 1990 and 2010, the population aged ≥60 years increased by 75.1% [10]. A World Health Organization (WHO) report (1998) also reports that 5.6% of Pakistan’s population was over 60 years of age, with a probability of doubling to 11% by the year 2025 [11]. With the lack of literature on drug utilization in older adults in the developing world and the rising global demographic of older adults, this points toward the need for drug utilization studies in this area. Drug utilization research is a valuable tool to guide health policy-makers in making their decisions. Similarly, this facilitates value-added communication amongst healthcare personnel, healthcare authorities and scientists [12]. Drug utilization research assesses the utilization and impact of medicines in the community and plays a key role in prioritizing the medical needs of a given country through guiding selection of medicines for national formularies. Reports from drug utilization reviews are important tools employed in the assessment of healthcare practices. The findings of drug utilization surveys also help to improve the rational use of medicines [13].

As such, the objective of this study was to investigate drug utilization patterns among elderly hospitalized patients on poly-pharmacy regimens in Punjab province, Pakistan. Furthermore, we evaluated the combined effect of various factors (for example, demographic, socioeconomic, and health-related factors) on patients on poly-pharmacy in a representative sample of hospitalized elderly patients.

## Methods

### Study design

A descriptive, non-experimental, cross-sectional study was carried out in six tertiary-care hospitals in the Punjab province of Pakistan, to evaluate drug utilization patterns among elderly hospitalized patients who were on poly-pharmacy regimens.

For this study, data was collected and evaluated according to the objectives of the study. Elderly patients who had been hospitalized for at least 3 days and who were on ≥5 medicines per day were included in this study. Nutritional supplements, other than vitamins and electrolytes were not considered to be drugs and were not recorded.

### Study settings

Six tertiary care hospitals (1: Bahawal Victoria Hospital (BVH), 2: Nishtar hospital, 3: Allied hospital, 4: Mayo hospital, 5: District Headquarter (DHQ) Sargodha, 6: Benazir Bhutto hospital) from different areas of the Punjab province were selected as research sites.

The choice of tertiary-care hospitals was made through systematic random sampling. There are 23 tertiary-care hospitals in the Punjab province [14]. Out of these, six were randomly selected using the random number generator function in Microsoft Excel, thus negating the potential for selection bias. In Pakistan, the tertiary care hospitals are very similar in terms of staff and operations and consequently physicians follow the same prescribing practices. Similarly, the patient population is likely to be the same in tertiary care hospitals. Thus randomly selecting patients from these six hospitals is not expected to create issues with significant bias.

### Study population and sample size

The population under study were patients of age ≥ 60 years, who were hospitalized in six selected tertiary-care hospitals. According to the latest Pakistani Census, the population of the surveyed province consists of 91,379,615 individuals [15]. The minimum required sample size was 385, as calculated using the Raosoft sample size calculator [16], with 95% confidence interval (CI) and 5% margin of error [Eq. 1].

1 $$ n\kern0.5em =\kern0.5em Nx/\left(\left(N-1\right)E2\kern0.5em +\kern0.5em x\right) $$

Where N is the population size, x is the CI and E is the margin of error.

The study was conducted on the patients who were admitted in selected hospitals due to complications associated with their chronic illness rather than on the patients who were admitted due to an acute episode unrelated to their chronic condition.

### Data collection

Over a 3 month period (15 December 2015 to 14 March 2016), a total of 3129 elderly patients were approached in-order to obtain consent from 600 to participate. Data was collected at different intervals from these tertiary-care hospitals irrespective of the date of admission of patients.

A data collection form was designed [Additional file 1], which consisted of four main parts: demographic, socio-economic, health-related characteristics and drug utilization patterns. The reliability and internal consistency of the data collection form was assessed by conducting a pilot study. Piloting was undertaken using data from 60 patients. After piloting, the data collection form was restructured by adding chronic conditions and an area for a list of prescribed medicines, which was not part of the original form. The Cronbach’s alpha value was 0.84 demonstrating excellent reliability.

### Measurements

#### Demographic characteristics

The following categorical variables were recorded; gender (male/female), age (60–74, 75–89, ≥90 years), and civil status (single, married, divorced, widowed).

#### Socioeconomic characteristics

Education level (primary, secondary and tertiary), annual income (low, middle, upper class), residence (rural, urban), employment status (employed, unemployed) were the four variables measuring the socio-economic status of participants. Those participants who were retired (taking a pension) or running a business were classified as employed. The data was obtained through face to face questioning of all 600 patients.

#### Health-related characteristics

In-patient charts/medical records were used to collect this data. However, if more information on socio-demographic or health-related characteristics were needed, then patients or caregivers were interviewed. Health-related characteristics included the following; self-reported health (good, moderate, poor), health risks (smoking, alcohol consumption, obesity, none), health service utilization [normal clinic visits (≤3/year), high clinic visits (≤4/year)], and chronic diseases (heart diseases, respiratory, gastrointestinal, diabetes mellitus, joint diseases, hypertension, central nervous system (CNS) disorders, others) and comorbidities (present, absent). Obesity was assessed by the body mass index (BMI), and respondents were regarded as either normal (BMI < 25 kg/m^2^), overweight (25 ≤ BMI < 30 kg/m^2^) or obese (BMI ≥ 30 kg/m^2^) [1].

#### Drug utilization evaluation

All medicines in each prescription were noted on the pro-forma sheet. For the evaluation of drug utilization patterns, all the medicines from the 600 in-patient charts were classified under the Anatomical Therapeutic Chemical (ATC) classification system [17]. Furthermore, the most commonly prescribed active substances were categorized according to trends in use; low (prescribed to <10% of the selected patients), medium (prescribed to ≥10% of the selected patients but <40%) and high (prescribed to >40% of the selected patients).

### Statistical analysis

Statistical Package for Social Sciences (IBM, SPSS Statistics for Windows, version 21.0. Armonk, NY: IBM Corp.) was used for data analysis. Simple linear regression analysis was adopted to determine the association between variables. Multiple linear regression analysis was then carried out for statistically significant variables from the univariate analysis to identify factors associated with increased drug use among elderly hospitalized patients who were on polypharmacy regimens [18]. The 95% CI, beta, standard error, and *p*-value were described for each factor. Pseudo R square values were included to describe the percentages of variance explained by the model. *P*-value < .05 was deemed to be statistically signifiant [18].

## Results

A total of 3129 elderly hospitalized patients in six tertiary-care hospitals were approached and 600 consented patients (response rate: 19.2%) were included according to the inclusion & exclusion criteria. The response rate was low due to the frailty of the patients and their associated medical conditions meant they often opted not to participate in the study.

Just over half (52.7%, *n* = 316) of the participants were male and 70.3% (*n* = 422) were 60–74 years of age. Over three-quarters (77%, *n* = 462) were married and most (86.8%, *n* = 521) had primary education level and where of low annual income (79.5%, *n* = 477). Three-quarters (74.5%, *n* = 447) were unemployed (or on pensions) and a little over one half (55.8%, *n* = 335) were domiciled rurally. Self-reported health was moderate in 61.3% (*n* = 368) and a similar percentage (62%, *n* = 372) had attended ≤3 clinic visits in the previous year. Just over one-third (37.8%, *n* = 227) were smokers and co-morbidity was present in over one half (54%, *n* = 324) of the patients (Table 1).

**Table 1:** Characteristics of hospitalized elderly population

Variables		Male (*n* = 316)	Female (*n* = 284)	Total (*n* = 600)
		*n* (%)	*n* (%)	*n* (%)
Age (years)	60–74	239 (75.6)	183 (64.4)	422 (70.3)
	75–89	57 (18)	76 (26.8)	133 (22.2)
	≥90	20 (6.3)	25 (8.8)	45 (7.5)
Civil Status	Single	15 (4.7)	6 (2.1)	21 (3.5)
	Married	284 (89.9)	178 (62.7)	462 (77)
	Widowed	13 (4.1)	78 (27.5)	91 (15.2)
	Divorced	4 (1.3)	22 (7.7)	26 (4.3)
Education level	Primary (≤10 years)	259 (82)	262 (92.3)	521 (86.8)
	Secondary (11–13 years)	54 (17.1)	21 (7.4)	75 (12.5)
	Tertiary (≥14 years)	3 (0.9)	1 (0.4)	4 (0.7)
Annual income	Low class (PKR0–299,999)	237 (75)	240 (84.5)	477 (79.5)
	Middle class (PKR300,000–999,999)	63 (19.9)	41 (14.4)	104 (17.3)
	Upper class (PKR ≥ 1,000,000)	16 (5.1)	3 (1.1)	19 (3.2)
Employment status	Employed	114 (36.1)	39 (13.7)	153 (25.5)
	Unemployed	202 (63.9)	245 (86.3)	447 (74.5)
Residence	Rural (an area outside of cities and towns)	177 (56)	158 (55.6)	335 (55.8)
	Urban (a city area considered as the inner city)	139 (44)	126 (44.4)	265 (44.2)
Self-reported health	Good	14 (4.4)	20 (7)	34 (5.7)
	Moderate	208 (65.8)	160 (56.3)	368 (61.3)
	Poor	94 (29.7)	104 (36.6)	198 (33)
Health Service Utilization	Clinic visits ≤3/year	207 (65.5)	165 (58.1)	372 (62)
	Clinic visits ≥4/year	109 (34.5)	119 (41.9)	228 (38)
Health Risks	Smoking	189 (59.8)	38 (13.4)	227 (37.8)
	Alcohol Consumption	5 (1.6)	0 (0)	5 (0.8)
	Obesity	48 (15.2)	166 (58.5)	214 (35.7)
	None	74 (23.4)	80 (28.2)	154 (25.7)
Co-morbidity	Present	163 (51.6)	161 (56.7)	324 (54)
	Absent	153 (48.4)	123 (43.3)	276 (46)
Number of drugs	5	251 (79.4)	204 (71.8)	455 (75.8)
	6	53 (16.8)	62 (21.8)	115 (19.2)
	7	11 (3.5)	15 (5.3)	26 (4.3)
	8	1 (0.3)	3 (1.1)	4 (0.7)

The most common chronic conditions among participants were; gastrointestinal (37.8%), hypertension (32%) and joint diseases (25.7%) (Table 2).

**Table 2:** Chronic conditions associated with elderly hospitalized patients on polypharmacy

Male (*n* = 316)		Female (*n* = 284)		Total (*n* = 600)	
Chronic conditions	*n* (%)	Chronic conditions	*n* (%)	Chronic conditions	*n* (%)
Heart diseases^a^	62 (19.6)	Heart diseases^a^	43 (15.1)	Heart diseases^a^	105 (17.5)
Respiratory^b^	74 (23.4)	Respiratory^b^	69 (24.3)	Respiratory^b^	143 (23.8)
Gastrointestinal^c^	123 (38.9)	Gastrointestinal^c^	104 (36.6)	Gastrointestinal^c^	227 (37.8)
Diabetes Mellitus	41 (13)	Diabetes Mellitus	71 (25)	Diabetes Mellitus	112 (18.7)
Joint diseases^d^	75 (23.7)	Joint diseases^d^	79 (27.8)	Joint diseases^d^	154 (25.7)
Hypertension	89 (28.2)	Hypertension	103 (36.3)	Hypertension	192 (32)
CNS disorders^e^	63 (19.9)	CNS disorders^e^	46 (16.2)	CNS disorders^e^	109 (18.2)
Others	46 (14.6)	Others	49 (17.3)	Others	95 (15.8)

^a^(Heart failure, Coronary ischemic disease, Atrial fibrillation, Stenosis)

^b^(Chronic bronchitis, Asthma, Chronic obstructive pulmonary disease)

^c^(Peptic ulcer, Irritable bowel syndrome)

^d^(Osteoarthritis, Rheumatoid arthritis)

^e^(Alzheimer’s disease, Epilepsy, Depression, Anxiety)

The most commonly prescribed drug classes were: A: alimentary tract and metabolism (80%), N: nervous system (66.3, J: anti-infectives for systemic use (62.2and C: cardiovascular system (48.3%), respectively. The detailed description about the drug utilization pattern is given in Tables 3 and 4.

**Table 3:** Prescription drug utilization in study participants

Prescription Drug Utilization (%)																		
Pharmacologic groups	60–74 years				75–89 years				≥90 years				Total					
	Male (*n* = 239)	%	Female (*n* = 183)	%	Male (*n* = 57)	%	Female (*n* = 76)	%	Male (*n* = 20)	%	Female (*n* = 25)	%	Male (*n* = 316)	%	Female (*n* = 284)	%	Overall (*n* = 600)	%
A = Alimentary tract and metabolism	182	76.2	151	82.5	44	77.2	58	76.3	20	100	25	100	246	77.8	234	82.4	480	80
B = Blood and blood-forming organs	25	10.5	19	10.4	3	5.3	6	7.9	1	5	2	8	29	9.2	27	9.5	56	9.3
C = Cardiovascular system	102	42.7	81	44.3	36	63.2	44	57.9	12	60	15	60	150	47.5	140	49.3	290	48.3
D = Dermatologic	10	4.2	7	3.8	8	14	7	9.2	2	10	2	8	20	6.3	16	5.6	36	6
G = Genitourinary system	3	1.3	0	0	0	0	0	0	0	0	0	0	3	0.9	0	0	3	0.5
H = Systemic hormonal agents	10	4.2	18	9.8	2	3.5	2	2.6	2	10	2	8	14	4.4	22	7.7	36	6
J = Anti-infectives for systemic use	147	61.5	133	72.7	25	43.9	40	52.6	13	65	15	60	185	58.5	188	66.2	373	62.2
L = Antineoplastic and immune-modulating agents	7	2.9	2	1.1	1	1.8	3	3.9	0	0	0	0	8	2.5	5	1.8	13	2.2
M = Musculoskeletal system	70	29.3	56	30.6	18	31.6	26	34.2	2	10	12	48	90	28.5	94	33.1	184	30.7
N = Nervous system	160	66.9	134	73.2	34	59.6	48	63.2	12	60	10	40	206	65.2	192	67.6	398	66.3
P = Antiparasitic products	8	3.3	1	0.5	0	0	2	2.6	1	5	0	0	9	2.8	3	1.1	12	2
R = Respiratory system	65	27.2	54	29.5	20	35.1	29	38.2	5	25	9	36	90	28.5	92	32.4	182	30.3
V = Various	0	0	3	1.6	0	0	1	1.3	0	0	0	0	0	0	4	1.4	4	0.7

**Table 4:** Drug utilization pattern in study participants (ATC level 1–4)

Drug Utilization pattern (%^a^)											
Level 1	*n*	%	Level 2	n	%	Level 3	*n*	%	Level 4	*n*	%
A	480	80	A01: Stomatological preparations	120	20	A01A: Stomatological preparations	120	20	A01AB: Anti-infectives and antiseptics for local oral treatment	120	20
			A02: Drugs for acid related disorders	387	64.5	A02B: Drugs for peptic ulcer and GERD	382	63.7	A02BA: H2-receptor antagonists	60	10
									A02BC: Proton pump inhibitors	309	51.5
			A03: Drugs for functional gastrointestinal disorders	129	21.5	A03F: Propulsives	116	19.3	A03FA: Propulsives	116	19.3
			A10: Drugs used in diabetes	117	19.5	A10B: Blood glucose lowering drugs	72	12	A10BB: Sulfonylureas	53	8.8
B	56	9.3	B01: Antithrombotic agents	26	4.3	B01A: Antithrombotic agents	26	4.3	B01AC: Platelet aggregation inhibitors	23	3.8
			B05: Blood substitutes	27	4.5	B05B:I.v. solutions	27	4.5	B05BC: Osmotic diuresis	27	4.5
C	290	48.3	C03: Diuretics	75	12.5	C03C: High-ceiling diuretics	68	11.3	C03CA: Sulfonamides, plain	68	11.3
			C07: Beta blocking agents	119	19.8	C07A: Beta blocking agents	119	19.8	C07AB: Beta blocking agents, selective	62	10.3
			C08: Calcium channel blockers	99	16.5	C08C: Selective calcium channel blockers	91	15.2	C08CA: Dihydropyridine derivatives	91	15.2
			C10: Lipid modifying agents	99	16.5	C10A: Lipid modifying agents, plain	97	16.2	C10AA: HMG CoA reductase inhibitors	97	16.2
D	36	6	D07: Corticosteroids	41	6.8	D07A: Corticosteroids, plain	41	6.8	D07AA: Corticosteroids, weak (group I)	30	5
G	3	0.5	G04: Urologicals	4	0.7	G04C: Drugs used in BPH	2	0.3	G04CA: Alpha-adrenoreceptor antagonists	2	0.3
H	36	6	H01: Pituitary and hypothalamic hormones	31	5.2	H01C: Hypothalamic hormones	31	5.2	H01CB: Somatostatin and analogues	31	5.2
J	373	62.2	J01: Antibacterials for systemic use	495	82.5	J01D: Other beta-lactam antibacterials	266	44.3	J01DD: 3rd-generation cephalosporins	255	42.5
						J01 M: Quinolone antibacterials	68	11.3	J01MA: Fluoroquinolones	68	11.3
			J04: Antimycobacterials	92	15.3	J04A: Drugs for tuberculosis	92	15.3	J04AK: Other drugs	48	8
L	13	2.2	L01: Antineoplastic agents	17	2.8	L01B: Antimetabolites	04	0.7	L01BA: Folic acid analogues	03	0.5
M	184	30.7	M01: Anti-inflammatory and antirheumatic products	188	31.3	M01A: Anti-inflammatory and antirheumatic products, non-steroids	188	31.3	M01AB: Acetic acid derivatives	159	26.5
N	398	66.3	N02: Analgesics	403	67.2	N02B: Other analgesics and antipyretics	359	59.8	N02BE: Anilides	305	50.8
			N03: Antiepileptics	67	11.2	N03A: Antiepileptics	67	11.2	N03AX: Other antiepileptics	32	5.3
P	12	2	P01: Antiprotozoals	13	2.2	P01B: Antimalarials	12	2	P01BF: Artemisinin and derivatives	9	1.5
R	182	30.3	R01: Nasal preparations	84	14	R01A: Decongestants and other	84	14	R01AD: Corticosteroids	61	10.2
			R03: Drugs for obstructive airway diseases	113	18.8	R03A: Adrenergics, inhalants	86	14.3	R03AC: Selective beta-2 agonists	43	7.2
			R06: Antihistamines for systemic use	67	11.2	R06A: Antihistamines for systemic use	67	11.2	R06AA: Aminoalkyl ethers	46	7.7
V	4	0.7	V03: Other therapeutic products	6	1	V03A: Other therapeutic products	6	1	V03AB: Antidotes	6	1

Note: A patient may be prescribed one or more than one drug from level 2, level 3 and level 4 categories

^a^Percentages given with respect to the total sample size of the patients. GERD Gastroesophageal reflux disease, BPH Benign prostate hyperplasia

The most commonly prescribed active substances were; A02BC01: omeprazole (*n* = 308, 51.3%), N02BE01: paracetamol (*n* = 305, 50.8%) and J01DD04: ceftriaxone (*n* = 241, 40.2%) (Table 5). A detailed description about the usage of all prescribed medicines can be found in Appendix.

**Table 5:** Top active substances prescribed to study participants (ATC level 5)

Name	ATC Code	Frequency (*n* = 3179)	Percentage^a^	Trend in use
Amlodipine	C08CA01	78	13	Medium
Aspirin	N02BA01	54	9	Low
Atenolol	C07AB03	62	10.3	Medium
Captopril	C09AA01	53	8.8	Low
Ceftriaxone	J01DD04	241	40.2	High
Dexamethasone	R01AD03	61	10.2	Medium
Diclofenac sodium	M01AB05	111	18.5	Medium
Furosemide	C03CA01	68	11.3	Medium
Lactulose	A06AD11	49	8.2	Low
Metformin	A10BA02	53	8.8	Low
Metoclopramide	A03FA01	86	14.3	Medium
Metronidazole	A01AB17	119	19.8	Medium
Omeprazole	A02BC01	308	51.3	High
Paracetamol	N02BE01	305	50.8	High
Simvastatin	C10AA01	63	10.5	Medium

^a^Percentages given with respect to the total sample size of patients

After adjusting the factors associated with increased drug use among elderly hospitalized patients who were on polypharmacy regimens in the univariate analysis, the factors which remained significant in the multiple linear regression were; male gender (95% CI −.205, −.006, *p* = .039, B = −.091), being divorced (95% CI −.604, −.136, *p* = .002, B = −.130) and the presence of comorbidity (95% CI .068, .267, *p* = .001, B = .144) (Table 6).

**Table 6:** Factors affecting number of prescribed drugs: multiple linear regression analysis

Variables	Unstandardized Coefficients	Standardized Coefficients	*p*-value	95.0% Confidence Interval for B	
	Std. Error	B		Lower Bound	Upper Bound
Male	.051	−.091	*.039*	−.205	−.006
Widowed	.073	−.008	.865	−.157	.132
Divorced	.119	−.130	*.002*	−.604	−.136
Low income class	.135	.051	.592	−.193	.339
Middle income class	.144	−.034	.716	−.335	.230
Moderate self-reported health	.105	−.075	.397	−.296	.118
Poor self-reported health	.127	−.034	.744	−.292	.208
≥4 clinic visits	.076	.075	.238	−.060	.240
Comorbidity	.051	.144	*.001*	.068	.267

*P*-value < .05 was considered statistically significant. Note: Only statistically significant variables in the univariate analysis were entered in the multiple linear regression analysis and are shown in the Table

Model summary: R^2^ = 0.052, *p* < 0.0005

## Discussions

This large pharmaco-epidemiological study set out to determine drug utilization patterns of elderly patients on poly-pharmacy regimens within six hospitals in Punjab province, Pakistan. Furthermore, the study evaluated the combined effect of factors including; demographic, socioeconomic, and health-related issues on poly-pharmacy in this population. This Discussion compares a summary of the findings with the literature and notes the contribution, outlines the implications for policy, practice and future research and considers the limitations of the study.

### Drug utilization patterns

Alimentary tract and metabolism category drugs were the most commonly prescribed class. Pakistan has been afflicted by alimentary tract disorders (ATDs) with an estimated prevalence of 45% [19]. It has been seen that ATDs affect a large number of people (approximately 60 to 70 million people in the US each year) and contribute substantially to morbidity and mortality [20]. According to one estimate, these disorders pose a significant fiscal and societal burden in the US [21]. In 2004 in the US, there were approximately 72 million ambulatory care visits, 4.6 million hospitalizations, 236,000 deaths and an estimated economic burden of $142 billion due to ATDs [22]. A study conducted in Finland reported that Alimentary tract and metabolism category drugs were prescribed to 77% of elderly patients who were on poly-pharmacy regimens [23]. Similarly, another study conducted in Italy reported that ATDs were prescribed to 42% of this population [24]. Thus the common prevalence of ATDs globally has led to increased utilization of alimentary tract and metabolism category drugs.

In the alimentary tract category, the most frequently prescribed sub-classes were; A02: drugs for acid related disorders (64.5%), A03: drugs for functional gastrointestinal disorders (21.5%), A01: stomatological preparations (20%) and A10: drugs used in diabetes (19.5%), respectively (Table 4). Drugs for acid related disorders are most commonly prescribed because they are generally safe and effective medicines used to treat gastric ulcers, heartburn, and gastro-oesophageal reflux disease (GORD). In this category, proton pump inhibitors (PPIs) are the highest-selling drugs worldwide. In addition to the treatment of gastritis, proton pump inhibitors are also very commonly prescribed as a gastro-protectant for patients prescribed antiplatelet and non-steriodal anti-inflammatory drugs (NSAIDs). In some countries they are available over-the-counter (OTC). Nexium (esomeprazole) in the US, earns nearly 6 billion USD and Risek (omeprazole) in Pakistan, earns close to 2 billion PKR, according to IMS Health data from 2012 [25]. It must be kept in mind that the chronic use of PPIs is associated with problems such as osteoporosis, hip fracture, escalated risk of infections, hypergastrinemia, decreased absorption of vitamins and minerals, kidney damage, dementia to name a few [26–28]. Healthcare professionals must adhere to prescribing guidelines and curb the excessive use of PPIs in-order to minimize associated adverse effects and reduce costs.

Nervous system drugs were the second most commonly prescribed class attributed to the high prevalence of neurologic and psychiatric disorders in this study. According to a World Health Organization Report, depression is the leading cause of health-related disability, globally [29]. Furthermore, a study on mood disorders in 30 European countries estimated that approximately 165 million elderly people (38% of the total population of these countries) suffer from significant mental illness [30]. In Pakistan, neurologic and psychiatric disorders are indeed prevalent [31]. A systematic review reported that the mean overall prevalence of anxiety and depressive disorders in the Pakistani population is 34% (range 29–66% for women and 10–33% for men) [32]. Three studies conducted in the Finnish elderly population reported that nervous system drugs were prescribed to between 63% and 89% in this group of patients [23, 33, 34]. Another study in nearby Sweden reported that this class was prescribed to 37% of the elderly population [35]. Thus neurological disorders afflicts both high income and low income countries with comparatively high treatment rates in high income countries [36].

In the nervous system category, frequently prescribed sub-classes in this study were; N02: Analgesics (67.2%), N03: Anti-epileptics (11.2%), respectively (Table 4). Analgesics, main therapy for low back pain, were the most frequently prescribed agents because low back pain is commonplace in the elderly, due to ageing of intervertebral discs [37, 38] with a prevalence of 60–70% in industrialized nations [39]. In 2010 Global Burden of Disease Study estimated that low back pain was one of the top 10 injuries and diseases throughout the world [40]. An American study reported that an estimated 149 million work days were lost due to low back pain, with an economic burden of USD 100 to 200 billion [41, 42].

The third most frequently prescribed drugs in this study were from the anti-infectives for systemic use class. These medicines are prescribed for a variety of infections caused by bacteria, virus, fungi, viroids, prions, nematodes, arthropods and so forth. A range of medicines are used to treat infections including; antivirals, antibiotics, antifungals, antihelminthics, and antiprotozoals. Infectious diseases accounted for 9.2 million deaths worldwide in 2013 (approximately 17% of all deaths) [43]. As in many other developing countries, infectious diseases are common in Pakistan and therefore anti-infectives are commonly prescribed and have a large market size [44]. A study conducted in Italy reported that anti-infectives for systemic use category drugs were prescribed to 41% of the elderly population [24]. Another study conducted in Sweden reported that anti-infectives were prescribed to just over one quarter (27.6%) of the elderly population [35]. It is likely that inter-country variability of infectious diseases is responsible for the varying patterns of global antibiotic use. In the Anti-infectives (for systemic use) category, the most frequently prescribed sub-classes were; J01: anti-bacterials for systemic use (82.5%) and J04: anti-mycobacterials (15.3%) (Table 4). Anti-bacterials/antibiotics are the most widely consumed pharmaceutical group, worldwide [45]. According to one estimate, the utilization of antibiotics has increased by 36% over the 10 years from 2000 to 2010. Russia, China, South Africa, India and Brazil are accountable for 76% of this of the prescribing and the growth [46]. In India, a rise from 29 to 57% was seen in Klebsiella pneumonia between 2008 and 2014. The concerning aspect here is that Klebseilla is becoming increasingly resistant to very potent antibiotics such as carbapenems [45]. The other concern is that this figure is considerably lower in the US and Europe i.e. less than 10% [45]. Generally, for most countries the usage of antibiotics varies with the season [46] and this appears to be no different in Pakistan.

The fourth most frequently prescribed medicines in this study were for cardiovascular diseases. These findings are in line with the fact that cardiovascular disorders (CVDs) are the most prevalent and leading causes of death worldwide [47], resulting in 17.3 million deaths in 2013 [43]. In 2010, the total costs of CVD globally was estimated to be in the vicinity of 315.4 billion USD [48]. Amongst the elderly population, 71% of people aged between 60 and 80 years, and 85% of people over 80 years are estimated to have CVD [49]. Similarly, in Pakistan, amongst the elderly population, 76% of people aged between 60 and 70 years, and 83% of people over 90 years are estimated to have CVD [50]. According to a Danish study of persons aged ≥70 years, cardiovascular drugs (35%) were the most frequently prescribed class [25]. A Danish study revealed that the most commonly prescribed medicines amongst 75 year olds were cardiovascular (25%) drugs [12] where it was almost double this in Sweden with 47% of elderly being prescribed cardiovascular drugs [41]. In the cardiovascular category, the most frequently prescribed sub-classes were; C07: beta blocking agents (19.8%), C08: calcium channel blockers (16.5%), C10: lipid-modifying agents (16.5%) and C03: diuretics (12.5%) respectively (Table 4). Beta-blocking agents are most widely prescribed because they are used to manage cardiac arrhythmias and myocardial infarction, as well as hypertension [51]. Diuretics were the most commonly prescribed class because they represent the first-line treatment for hypertension and the prevalence of hypertension is high throughout the world; it affected between 30 and 45% of the population of Europe in 2013 [52].

In summary, the most commonly prescribed active substances were; omeprazole, paracetamol and ceftriaxone. This follows the developed world with regards omeprazole and paracetamol but the excessive use of potent IV antibiotics such as ceftriaxone warrants serious review.

### Factors associated with “poly-pharmacy”

Results from the multiple linear regression analysis revealed that male gender (negatively associated); being divorced (negatively associated) and comorbidity (positively associated) were the main factors associated with increased drug use among elderly hospitalized patients in Pakistan, who were on poly-pharmacy regimens.

The greater ratio of female to male gender amongst these elderly hospitalized patients on poly-pharmacy regimens could be attributed to physiological aspects, which differentially adjust the etiology patterns for females and males. These altered etiology patterns may represent illness behaviors such as being more sensitive to their health and consequently taking more medicines. It is also possible that there are more women in hospital because men simply die at an earlier age, often without even getting to hospital [53]. Such differences explain the use of specific types of medicines by women only. Simultaneously, the societal roles adopted by women in Pakistan, principally as housewives or paid employees also influence these gender differences [43]. Numerous studies have proposed that multiple roles for women, including home-maker, parent, and paid employees are likely to be hectic and harmful to their health [54–56]. Civil status, such as ‘being divorced’ played an important role in the illness behavior in this study. Amongst this subgroup of the elderly, psychological conditions such as depression and anxiety contributed towards poly-pharmacy [56]. Comorbidities are a significant factor associated with poly-pharmacy and increased mortality in older people. The elderly are significantly more prone to comorbidities due to aging and physiological changes; the majority of the older people (up to about 80%) suffer from chronic illnesses [8]. Consequently, they are more likely to have greater drug utilization to manage their chronic illnesses [9].

### Implications for policy and practice

There are implications from the findings of this study for pharmaceutical policy and practice in Pakistan.

In terms of policy and practice, there has been very little evaluation of “poly-pharmacy” in the context of Pakistan and so this study contributes significantly to that understanding. This study raises the question of whether prescribing needs to change in some way? Omeprazole was the most commonly prescribed pharmaceutical, followed by paracetamol and this appears to follow the trends in developing countries. What is most concerning, is the very high use of powerful IV antibiotics such as ceftriaxone. A national policy and guidelines need to be put in place to ensure the rational prescribing of potent antibiotics such as this. Further, in Pakistan infrastructure for proper medicine dispensing and patient education is not available within the health system and the availability of pharmacists at public hospital and private pharmacies is negligible. Elderly people who are on polypharmacy regimens are at risk of medicines overuse and adverse effects associated with polypharmacy. There is a global trend to “de-prescribe” in the setting of hospitalized older adults and the government should take the appropriate measures to ensure skilled pharmacists are available to enact this.

### Implications for future research

Future research could look at evaluating the impact of pharmacists around implementing and monitoring drug use indicators and clinical guidelines. Further, studies like the current one could be extended to assess potential drug interactions, trends in prevalence and determinants of potentially inappropriate medication use based on the Beer’s Criteria among the elderly population. This study recruited hospitalized elderly patients and it cannot be assumed that the sample is representative in any form of the ambulatory primary care population. Studies need to be conducted in the general community to better understand pharmaco-epidemiological patterns across the wider population. It will be interesting to explore in more detail the impact of being divorced on medicines use in Pakistan as the data (un-expectantly) suggests this is a significant determinant.

### Study limitations

There are a few study limitations. First, the population under study was elderly patients being hospitalized in the selected tertiary-care hospitals. Second, only those elderly patients were approached who are taking more than five prescribed medicines per day. Third, DDDs for the prescribed medicines to the hospitalized elderly patients were not calculated. Finally, the study did not investigate the ADRs associated with poly-pharmacy. However, this study provides the baseline information to the researchers regarding the drug utilization pattern among a cohort of hospitalized elderly patients in the Punjab province of Pakistan.

## Conclusion and recommendations

The increased use of prescription medicines is commonplace amongst the elderly population worldwide, and this study suggests it is no different in Pakistan. Similarly, the average number of medicines being used by elderly women is on the rise. This study concludes that a series of factors are responsible for “poly-pharmacy” in older adults in Pakistan including; being male, being divorced and the presence of multiple comorbidities. The most common chronic conditions associated with these hospitalized elderly patients were; gastrointestinal, hypertension and joint diseases, respectively. In this study, the most commonly prescribed drug classes reflect that seen in developed nations including; Alimentary tract and metabolism, Nervous system, Antibacterial for systemic use and Cardiovascular system, respectively.

There is no doubt that the usage of medicines is essential; however, poly-pharmacy is likely to also compound in a cyclical manner the associated problems with the use of multiple medicines. It is highly recommended that greater consideration be given to elderly hospitalized patients who are on poly-pharmacy regimens, in Pakistan. To reduce the potential for ADRs associated with poly-pharmacy and to get the maximum benefit of therapy, there is a requirement to use medicines effectively and to evaluate progress at regular intervals based on diagnosis and expected treatment outcomes. The rational use of medicines is of utmost importance, most particularly in the elderly population and the responsibility lies with healthcare professionals to regularly evaluate medicine use.

Unfortunately, geriatrics is not recognized as a specialized area of research in Pakistan and hospital pharmacists are not as commonplace; as they are in developed nations. Under the current scenario, the role of the pharmacist in the management of elderly patients must be enhanced in Pakistan and the impact of this intervention could be tested by employing experimental study designs. The contribution of this paper is that it provides baseline information about the prescribing patterns in the older hospitalized patients in Pakistan and provides a platform for evaluation of policy and practice interventions by Pakistani hospital pharmacists in the future.

## Appendix

**Table 7:** Description about the usage of all prescribed medicines

Sr. No	Name	ATC Code	Frequency (*N* = 3179)	Percentage^a^	Trend
1	Aciclovir	J05AB01	11	1.8	Low
2	Acarbose	A10BF01	1	0.2	Low
3	Aclarubicin	L04AB04	1	0.5	Low
4	Adalimumab	L01DB04	3	0.2	Low
5	Alimemazine	R06AD01	2	0.3	Low
6	Allopurinol	M04AA01	17	2.8	Low
7	Amantadine	N04BB01	1	0.2	Low
8	Amiodarone	C01BD01	2	0.3	Low
9	Aminophylline	R03DA05	16	2.7	Low
10	Amitriptyline	N06AA09	2	0.3	Low
11	Amoxicillin	J01CA04	27	4.5	Low
12	Amlodipine	C08CA01	78	13.0	Medium
13	Anastrazole	L02BG03	2	0.3	Low
14	Artemether	P01BF01	08	1.3	Low
15	Aspirin	N02BA01	54	9.0	Low
16	Atenolol	C07AB03	62	10.3	Medium
17	Atropine	A03BA01	4	0.7	Low
18	Atorvastatin	C10AA05	15	2.5	Low
19	Attapulgite	A07BC04	1	0.2	Low
20	Azithromycin	J01FA10	13	2.2	Low
21	Beclomethasone	D07AC15	11	1.8	Low
22	Bisoprolol	C07AB07	1	0.2	Low
23	Bismuth subcitrate	A07BX05	1	0.2	Low
24	Benzyl penicillin	J01 CE01	7	1.2	Low
25	Bleomycin	L01 DC01	1	0.2	Low
26	Bromazepam	N05BA08	8	1.3	Low
27	Bromocriptine	G02CB01	3	0.5	Low
28	Calcium gluconate	A12AA03	1	0.2	Low
29	Captopril	C09AA01	53	8.8	Low
30	Carbamazepine	N03AF01	6	1.0	Low
31	Carvedilol	C07AG02	2	0.3	Low
32	Cefotaxime	J01DD01	14	2.3	Low
33	Ceftriaxone	J01DD04	241	40.2	High
34	Celecoxib	M01AH01	5	0.8	Low
35	Cephradine	J01DB09	4	0.7	Low
36	Cetirizine	R06AE07	2	0.3	Low
37	Cimetidine	A02BA01	3	0.5	Low
38	Chlorhexidine	A01AB03	1	0.2	Low
39	Chloramphenicol	J01BA01	6	1.0	Low
40	Chlorpheniramine	R06AB04	11	1.8	Low
41	Chlorpromazine	N05AA01	6	1.0	Low
42	Colecalciferol	A11CC05	6	1.0	Low
43	Ciprofloxacin	J01MA02	34	5.7	Low
44	Clarithromycin	J01FA09	25	4.2	Low
45	Clindamycin	J01FF01	7	1.2	Low
46	Clopidogrel	B01AC04	23	3.8	Low
47	Clonazepam	N03AE01	2	0.3	Low
48	Clonidine	C02AC01	2	0.3	Low
49	Codeine	R05DA04	2	0.3	Low
50	Colchicine	M04 AC01	21	3.5	Low
51	Cromolyn sodium	A07EB01	3	0.5	Low
52	Cyclosporine	N04 AD01	2	0.3	Low
53	Dacarbazine	L01AX04	2	0.3	Low
54	Darifenacin	G04BD10	1	0.2	Low
55	Drotaverine	A03AD02	5	0.8	Low
56	Dexamethasone	R01AD03	61	10.2	Medium
57	Diclofenac sodium	M01AB05	111	18.5	Medium
58	Dicyclomine	A03AA07	1	0.2	Low
59	Digoxin	C01AA05	3	0.5	Low
60	Diphenhydramine	R06AA02	46	7.7	Low
61	Diphenoxylate	A07DA01	3	0.5	Low
62	Diloxanide	P01AC01	1	0.2	Low
63	Diltiazem	C08DB01	1	0.2	Low
64	Divalproex sodium	N03AG01	25	4.2	Low
65	Dobutamine	C01CA07	2	0.3	Low
66	Domperidone	A03FA03	30	5.0	Low
67	Dopamine	C01CA04	2	0.3	Low
68	Doxycycline	J01AA02	2	0.3	Low
69	Epinephrine	B02BC09	4	0.7	Low
70	Ephedrine	R01AA03	1	0.2	Low
71	Erythromycin	J01FA01	1	0.2	Low
72	Escitalopram	N06AB04	18	2.8	Low
73	Ethambutol	J04AK02	25	4.2	Low
74	Ezitimibe	C10AX09	2	0.3	Low
75	Famotidine	A02BA03	20	3.3	Low
76	Fexofenadine	R06AX26	6	1.0	Low
77	Fluoxetine	N06AB03	2	0.3	Low
78	Formoterol	R03AC13	4	0.7	Low
79	Fosfomycin	J01XX01	4	0.7	Low
80	Furosemide	C03CA01	68	11.3	Medium
81	Gentamicin	J01GB03	9	1.3	Low
82	Glimepiride	A10BB12	10	1.7	Low
83	Glipizide	A10BB07	9	1.5	Low
84	Glyceryl trinitrite	C01DA02	9	1.5	Low
85	Haloperidol	N05 AD01	5	0.8	Low
86	Heparin	B01AB01	3	0.5	Low
87	Hydrocortisone	D07AA02	30	5.0	Low
88	Hydralline	C02DB02	15	2.5	Low
89	Hydralazine	C02DB02	6	1.0	Low
90	Ibuprofen	M01AE01	24	4.0	Low
91	Imipenem	J01DH51	3	0.5	Low
92	Imipramine	N06AA02	1	0.2	Low
93	Indacaterol	R03AC18	2	0.3	Low
94	Indomethacin	M01AB01	5	0.8	Low
95	Insulin	A10AB02	40	6.7	Low
96	Ipratropium	R01AX03	19	3.2	Low
97	Isoniazid	J04 AC01	23	3.8	Low
98	Isosorbide mononitrite	C01DA14	3	0.5	Low
99	Isosorbide dinitrite	C01DA08	2	0.3	Low
100	Ketorolac	M01AB15	43	7.2	Low
101	Lactulose	A06AD11	49	8.2	Low
102	Labetalol	C07AG01	3	0.5	Low
103	Leflunamide	L04AA13	3	0.5	Low
104	Leviteracetam	N03AX14	32	5.3	Low
105	Levodopa	N04BA01	4	0.7	Low
106	Levofloxacin	J01MA12	11	1.8	Low
107	Lisinopril	C09AA03	8	1.3	Low
108	Loperamide	A07DA03	5	0.8	Low
109	Losartan	C09CA01	3	0.5	Low
110	Lovastatin	C10AA02	2	0.3	Low
111	Lumefantrine	P01BF01	1	0.2	Low
112	Mannitol	B05BC01	27	4.5	Low
113	Mebeverine	A03AA04	3	0.5	Low
114	Mecobalamine	B03BA01	2	0.3	Low
115	Midazolam	N05CD08	3	0.5	Low
116	Mercaptopurine	L01BB02	1	0.2	Low
117	Meropenem	J01DH02	4	0.7	Low
118	Metformin	A10BA02	53	8.8	Low
119	Methotrexate	L01BA01	3	0.5	Low
120	Metoclopramide	A03FA01	86	14.3	Medium
121	Metoprolol	C07AB02	1	0.2	Low
122	Metaproterenol	R03AB03	1	0.2	Low
123	Metronidazole	A01AB17	119	19.8	Medium
124	Midazolam	N05CD08	15	2.5	Low
125	Mirtazapine	M06AX11	3	0.5	Low
126	Misoprostol	A02BB01	4	0.7	Low
127	Mg-hydroxide	A02AA04	5	0.8	Low
128	Montelukast sodium	R03DC03	16	2.7	Low
129	Morphine	N02AA01	3	0.5	Low
130	Moxifloxacin	J01MA14	23	3.8	Low
131	Nalbufine	N02AF02	13	2.2	Low
132	Naltrexone	V03AB30	6	1.0	Low
133	Natalizumab	L04AA23	1	0.2	Low
134	Nedocromil	R01AC07	3	0.5	Low
135	Nefazodone	N06AX06	1	0.2	Low
136	Nifedipine	C08CA05	2	0.3	Low
137	Nimodipine	C08CA06	11	1.8	Low
138	Nitroglycerine	C01DA02	6	1.0	Low
139	Nitropruside	C02DD01	1	0.2	Low
140	Nitrazepam	N05CD02	2	0.3	Low
141	Octreotide	H01CB02	30	5.0	Low
142	Omeprazole	A02BC01	308	51.3	High
143	Paracetamol	N02BE01	305	50.8	High
144	Paclitaxel	L01CD01	3	0.5	Low
145	Pantoprazole	A02BC02	1	0.2	Low
146	Phenelzine	N06AF03	2	0.3	Low
147	Phenytoin	N03AB02	2	0.3	Low
148	Pipracilline	J01CA12	1	0.2	Low
149	Prazosin	C02CA01	2	0.3	Low
150	Prednisone	H02AB07	1	0.2	Low
151	Prednisolone	H02AB06	1	0.2	Low
152	Probenecid	M04AB01	3	0.5	Low
153	Procainamide	C01BA02	1	0.2	Low
154	Propoxyphene	N02 AC04	2	0.3	Low
155	Propranolol	C07AA05	20	3.3	Low
156	Propylthiouracil	H03BA02	3	0.5	Low
157	Polymixin	J01XB02	2	0.3	Low
158	Pyrazinamide	J04AK01	23	3.8	Low
159	Pyridoxine	A11HA02	17	2.8	Low
160	Quinine	P01BC01	3	0.5	Low
161	Ranitidine	A02BA02	37	6.2	Low
162	Rifampicin	J04AB02	21	3.5	Low
163	Rifaxamin	D06AX11	5	0.8	Low
164	Reserpine	C02AA02	1	0.2	Low
165	Rituximab	L01XX21	1	0.2	Low
166	Rosuvastatin	C10AA07	17	2.8	Low
167	Salbutamol	R03AC02	43	7.2	Low
168	Sulbactum	J01CG01	11	1.8	Low
169	Salmeterol	R03AC12	22	3.7	Low
170	Simvastatin	C10AA01	63	10.5	Medium
171	Sitagliptin	A10BH01	4	0.7	Low
172	Selegiline	N04BD01	2	0.3	Low
173	Sertraline	N06AB06	1	0.2	Low
174	Somatostatin	H01CB01	1	0.2	Low
175	Solifenacin	G04BD08	1	0.2	Low
176	Spironolactone	C03DA01	7	1.2	Low
177	Streptomycin	J01GA01	4	0.7	Low
178	Sucralfate	A02BX02	9	1.5	Low
179	Sodium picosulfate	A06AB08	1	0.2	Low
180	Sulfasalazine	A07EC01	3	0.5	Low
181	Tamsulosin	G04CA02	2	0.3	Low
182	Tazobactum	J01CG02	19	3.2	Low
183	Theophylline	R03DA04	5	0.8	Low
184	Thioridazine	N05 AC02	1	0.2	Low
185	Thyroxin	H03AA01	4	0.7	Low
186	Tizinidine	M03BX02	1	0.2	Low
187	Terbutaline	R03AC03	4	0.7	Low
188	Tramadol	N02AX02	26	4.3	Low
189	Transemic acid	B02AA02	7	1.2	Low
190	Telmisartan	C09CA07	5	0.8	Low
191	Trustuzumab	L01XC03	2	0.3	Low
192	Vancomycin	J01XA01	23	3.8	Low
193	Venlafaxine	N06AX16	1	0.2	Low
194	Verapamil	C08DA01	7	1.2	Low
195	Vincristine	L01CA02	1	0.2	Low
196	Ziprasidone	N05AE04	1	0.2	Low
197	Zinc sulphate	A12CB01	1	0.2	Low

^a^Percentages given with respect to total sample size (*n* = 600)

## Additional file

Additional file 1: Data collection form. (DOCX 22 kb)

## Abbreviations

ADRs: Adverse Drug Reactions
ATC: Anatomical Therapeutic Chemical
ATDs: Alimentary Tract Disorders
BMI: Body Mass Index
CNS: Central Nervous System
CVDs: Cardiovascular Disorders
DDDs: Defined Daily Doses
LBP: Low Back Pain
SPSS: Statistical Package for Social Sciences
WHO: World Health Organization
