# Supplementary material for: Drug utilization patterns among elderly hospitalized patients on poly-pharmacy in Punjab, Pakistan
Source: J Pharm Policy Pract. 2017 Aug 4;10:23. doi: 10.1186/s40545-017-0112-z (PMC5545050; doi:10.1186/s40545-017-0112-z)
Supplement: Additional file 1: — Data collection form. (DOCX 22 kb) [file 40545_2017_112_MOESM1_ESM.docx]

**Drug utilization pattern among heavy drug users in an elderly hospitalized population in Punjab, Pakistan:** **association with demographic, socio-economic and health-related characteristics**

Explanatory statement

I am the student of M.Phil. (Pharmacy Practice) from “The Islamia University of Bahawalpur”. For completion of my M.Phil. research work, I want to evaluate the “drug utilization pattern among heavy drug users in an elderly hospitalized population in Punjab, Pakistan”. I’ll be extremely grateful for your participation in this study. Your responses will only be shared with research fellows and I’ll ensure that any information in my report does not identify you as the respondent.

Name: City:

**Section 1: Demographic characteristics**

1. Gender i. Male ii. Female

1. Age (years) i. 60-74 ii. 75-89 iii. ≥90
2. Civil Status i. Single ii. Married iii. Widowed iv. Divorced

**Section 2: Socio-Economic Characteristics**

1. Education level i. Primary (≤10 years) ii. Secondary (11-13 years)

iii. Tertiary (≥14 years)

1. Annual income i. Low class (PKR0-299,999) ii. Middle class (PKR300,000 -999,999) iii. Upper class (PKR≥1,000,000)

1. Employment Status i. Employed ii. Unemployed

1. Residence i. Rural ii. Urban

**Section 3: Health-Related Characteristics**

1. Self-reported health i. Good ii. Moderate iii. Poor
2. Health Service Utilization i. Normal clinic visits (≤3/year)

ii. High clinic visits (≤4/year)

1. Health Risks i. Smoking ii. Alcohol Consumption iii. Obesity

iv. None

1. Co-morbidity i. Present ii. Absent
2. Please indicate which chronic condition(s) you have:

i. Heart diseases* ii. Respiratory** iii. Gastro-intestinal***

iv. Diabetes Mellitus (I & II) v. Joint diseases**** vi. Hypertension

vii. CNS disorders***** viii. Others

* (Heart failure, Coronary ischemic disease, Atrial fibrillation, Stenosis)

** (Chronic bronchitis, Asthma, Chronic obstructive pulmonary disease)

*** (Peptic ulcer, Irritable bowel syndrome)

**** (Osteoarthritis, Rheumatoid arthritis)

***** (Alzheimer’s disease, Epilepsy, Depression, Anxiety)

**Section 4: Drug utilization pattern**

1. The drugs you are taking belongs to which category(ies)?

i. Alimentary tract/metabolism ii. Blood/blood forming organs

iii. Cardiovascular iv. Dermatologicals

v. Genito-urinary system/sex hormones vi. Systemic hormones

vii. Anti-infectives for systemic use viii. Musculoskeletal

ix. Antineoplastic and immunomodulating agents x. Nervous system

xi. Antiparasitic products xii. Respiratory system

Thank you for your help!

**Medicines Recommended:**
